# Genome-wide search identified DNA methylation sites that regulate the metabolome

**DOI:** 10.3389/fgene.2023.1093882

**Published:** 2023-05-18

**Authors:** Majid Nikpay

**Affiliations:** Omics and Biomedical Analysis Core Facility, University of Ottawa Heart Institute, Ottawa, ON, Canada

**Keywords:** DNA methylation, metabolite, biomarker, Mendelian randomization, epigenomics, metabolimics

## Abstract

**Background:** Identifying DNA methylation sites that regulate the metabolome is important for several purposes. In this study, publicly available GWAS data were integrated to find methylation sites that impact metabolome through a discovery and replication scheme and by using Mendelian randomization.

**Results:** The outcome of analyses revealed 107 methylation sites associated with 84 metabolites at the genome-wide significance level (*p*<5e^−8^) at both the discovery and replication stages. A large percentage of the observed associations (85%) were with lipids, significantly higher than expected (*p* = 0.0003). A number of CpG (methylation) sites showed specificity e.g., cg20133200 within *PFKP* was associated with glucose only and cg10760299 within *GATM* impacted the level of creatinine; in contrast, there were sites associated with numerous metabolites e.g., cg20102877 on the 2p23.3 region was associated with 39 metabolites. Integrating transcriptome data enabled identifying genes (N = 82) mediating the impact of methylation sites on the metabolome and cardiometabolic traits. For example, *PABPC4* mediated the impact of cg15123755-HDL on type-2 diabetes. *KCNK7* mediated the impact of cg21033440-lipids on hypertension. *POC5, ILRUN, FDFT1,* and *NEIL2* mediated the impact of CpG sites on obesity through metabolic pathways.

**Conclusion:** This study provides a catalog of DNA methylation sites that regulate the metabolome for downstream applications.

## Introduction

Metabolism is the process through which the body converts food into energy and materials necessary to maintain life. At the molecular level, it is conducted by a network of metabolites and is regulated in part by genomic regions known as epigenomic sites. Understanding the relationship between epigenome and metabolome is important for several purposes.

Imbalances in the level of a metabolite could lead to health abnormalities. Designing medications for metabolites is one approach to correct metabolic abnormalities; however, this approach is cumbersome (Sharma et al., 2021). A common workaround is to target the underlying epigenomic sites using the newly developed CRISPR-based techniques that enable precise epigenome editing (Nakamura et al., 2021). In this context, it is essential to know the epigenome sites that regulate the level of a metabolite. Furthermore, it is important to know the phenotypical consequences of editing a site, as well as the molecular path through which an epigenomic site exerts its impact.

Complex disorders are age-dependent and develop over time as abnormal changes at the molecular level accumulate. An important mechanism in this regard is epigenomics and tracking changes at epigenome sites is considered as a means to diagnose and prevent a disease, from an earlier stage (Napoli et al., 2012). Besides, epigenomic changes are reversible; therefore, if an abnormality is detected, a change in lifestyle can provide a remedy to offset harmful epigenomic changes and maintain the body in a healthy state (Bacalini et al., 2014; Quach et al., 2017).

Considering the above rationales, this study was devised to investigate epigenome sites that regulate the metabolome. The relationship between epigenomics and metabolomics has been the subject of a number of studies in the past (Frazier-Wood et al., 2014; Rodriguez et al., 2016; Zaghlool et al., 2018; Gomez-Alonso et al., 2021). The common approach in these studies is to measure the DNA-methylation levels and metabolites in the same group of individuals in order to identify CpG site-metabolite pairs that show correlation; however, such a design cannot tell whether a significant association indicates causation (CpG site → metabolite), correlation (CpG site ← confounders → metabolite) or reverse causation (metabolite → CpG site). Furthermore, limited sample sizes hinder the power of such studies. In recent years, a new approach called two-sample Mendelian randomization has been developed that allows integrating data from independent large GWAS consortia to investigate relations between biological features (biomarkers, phenotypes) (Davey Smith and Hemani, 2014; Zhu et al., 2018). In the current work, this approach was used to integrate GWAS data for DNA methylation sites and metabolites in order to find CpG sites that have casual impacts on the metabolites. The analyses were further extended to eQTLs and GWAS data for cardiometabolic traits to obtain functional insights.

## Materials and methods

### Data

This study was conducted using data from previous GWAS studies that made their findings publicly available. To make data comparable and control for population stratification, the search was limited to studies carried out in European populations. The majority of previous GWAS studies were conducted using blood samples and blood appears to be a good proxy for other tissues (Liu et al., 2017); therefore, only studies that used blood specimens were considered for analysis.

Based on these criteria, I obtained summary association statistics for SNPs regulating methylation sites from McRae et al. (McRae et al., 2018) in which the authors measured DNA methylation using Illumina HumanMethylation450 BeadChips in peripheral blood lymphocytes obtained from 1,980 subjects. GWAS summary statistics for metabolites were obtained from Julkunen et al. study (Julkunen et al., 2021) in which the authors used nuclear magnetic resonance spectroscopy to measure metabolites in plasma samples of 105,146 individuals obtained from the United Kingdom Biobank. GWAS summary statistics for a total of 248 blood metabolites were available from this study which includes 12 amino acids, 7 carbohydrates, 223 lipids, and 6 proteins.

mQTL data from Hannon et al. (Hannon et al., 2016; Hannon et al., 2018) were used to replicate the findings from the discovery stage. Here, the authors used Illumina HumanMethylation450 BeadChips to measure methylation levels in DNA samples extracted from whole blood in two separate studies. To gain functional/clinical insights, eQTL summary statistics for 19,942 genes were obtained from the eQTLGen consortium which represents a meta-analysis of 37 studies (a total of 31,684 individuals) conducted using blood samples. In addition, GWAS summary statistics for major cardiometabolic conditions including, coronary artery disease (CAD) (van der Harst and Verweij, 2018), body mass index (BMI) (Pulit et al., 2018), type 2 diabetes (T2D) (Mahajan et al., 2018), and hypertension (Source: United Kingdom Biobank) were obtained by surveying the recently published studies.

### Mendelian randomization

In the current study, the GSMR algorithm (Zhu et al., 2018) implemented in GCTA software (version 1.92) (Yang et al., 2011) was used to conduct Mendelian randomization (MR). As compared to other methods for MR analysis, GSMR automatically detects and removes SNPs that have a pleiotropic effect on both exposure and outcome; in addition, it accounts for the sampling variance in beta estimates and the linkage disequilibrium (LD) among SNPs. MR analysis uses a set of SNPs known as the instrument to test the relationship between exposure and outcome. Each set of SNPs must meet a number of criteria:

a) SNPs must not be in LD. In this study, I used SNPs that are in linkage equilibrium (*r* ^2^ < 0.05) based on genotype data from the 1000 genomes (n = 503 individuals of European ancestry).
b) SNPs included in MR analysis must not show pleiotropic effect (i.e., Exposure ← SNP → Outcome). Pleiotropic SNPs were excluded from MR using the HEID test implemented in GCTA software.
c) Each SNP must be significantly associated with the exposure. Only SNPs that were associated with the exposure at the GWAS significance level (P<5e^−8^) were selected for MR analysis.

### Analysis plan

**FIGURE 1:**
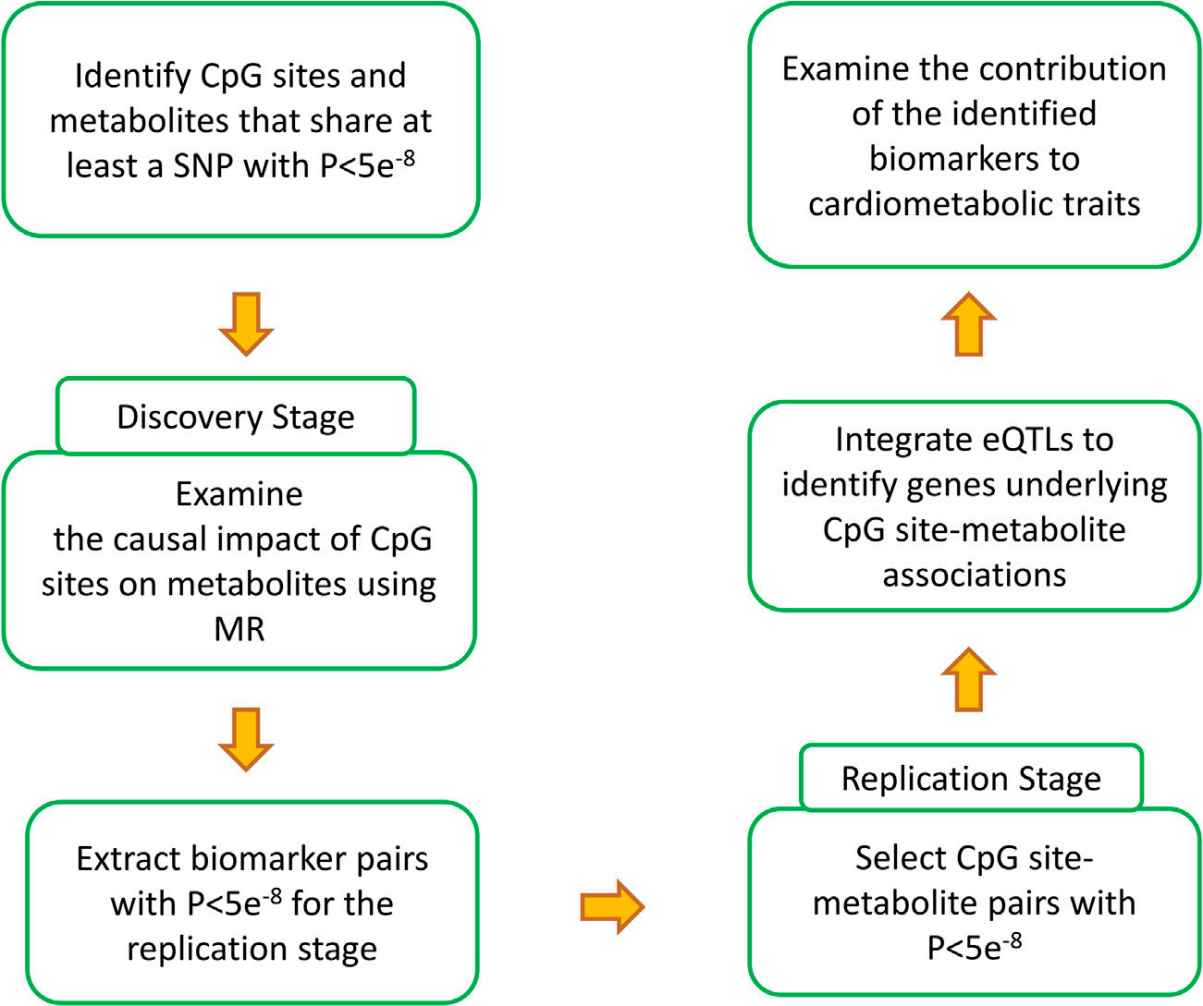
Summary of the analyses performed in the current study to generate the results. Through a discovery and a replication stage, CpG site-metabolite pairs that shared at least a SNP and showed causality (please see the methods section for details) were identified. Integrating eQTL data provided the possibility to investigate the intermediary genes. Finally, by integrating the identified biomarkers with GWAS data for cardimetabolic traits, a search was conducted to identify CpG sites that impact these traits through metabolic pathways and to investigate the underlying molecular mechanisms.

Figure 1 provides an overview of the analyses performed in this study. The first step was to find epigenomic-metabolic biomarkers that share at least a SNP associated with both at GWAS significance level (P<5e^−8^). In this manner, 399,944 CpG site-metabolite pairs were identified. Next, Mendelian randomization was used to examine whether the change of methylation at a site contributes to a metabolite. 3,0821 CpG site-metabolite pairs reached GWAS significance that were selected for the replication stage.

Secondary independent mQTL data were used to replicate the findings from the discovery stage. 1,105 pairs of epigenomic-metabolite biomarkers reached GWAS significance. To exclude the possibility of reverse causation (metabolite → CpG site, *p*<=0.05), then I swapped the places of exposure and outcome and re-examined the associations. Finally, given the complex pattern of linkage disequilibrium within the HLA region, CpG sites mapped to this region were also excluded from the results. Following this scheme, a total of 553 biomarker pairs that reached GWAS significance level (*p*-value < 5e^−8^) at both discovery and replication stages remained which consist of 107 CpG sites associated with 84 metabolites.

To obtain functional insight, then I integrated eQTL data from the eQTLGen consortium (Võsa et al., 2018) and used MR analysis to find genes that mediated the impact of methylation sites on metabolites. Namely, I looked for genes that met the condition:

CpG site → gene expression → metabolite

Following MR analysis, 82 genes were identified that mediated the impact of CpG sites on metabolites. Genes were then entered into the STRING (Search Tool for the Retrieval of Interacting Genes/Proteins) database (Version 11.5) (Szklarczyk et al., 2018) to find whether they are functionally related. Finally, for the purpose of clinical insight, a Unix package was devised that allows investigating the contribution of the identified biomarkers to a phenotype. In the following section, I describe the package.

### Unix package

Phenome is vast and diverse, and examining associations between the biomarkers identified in this study and each phenotype is not practical. Therefore, findings from this study is presented as a Unix package that allows a researcher to investigate the contribution of the identified biomarkers to a phenotype by specifying its identifier from the OpenGWAS database (Version 6.2.0), a repertoire of GWAS summary data for various phenotypic features (Elsworth et al., 2020). The underlying algorithm that carries out the task is written in the shell scripting language. This allows the use of parallel computing and therefore the possibility to examine a large volume of data. A guide on how to use the package is provided through the corresponding GitHub page (please see the data availability section).

## Results

**FIGURE 2:**
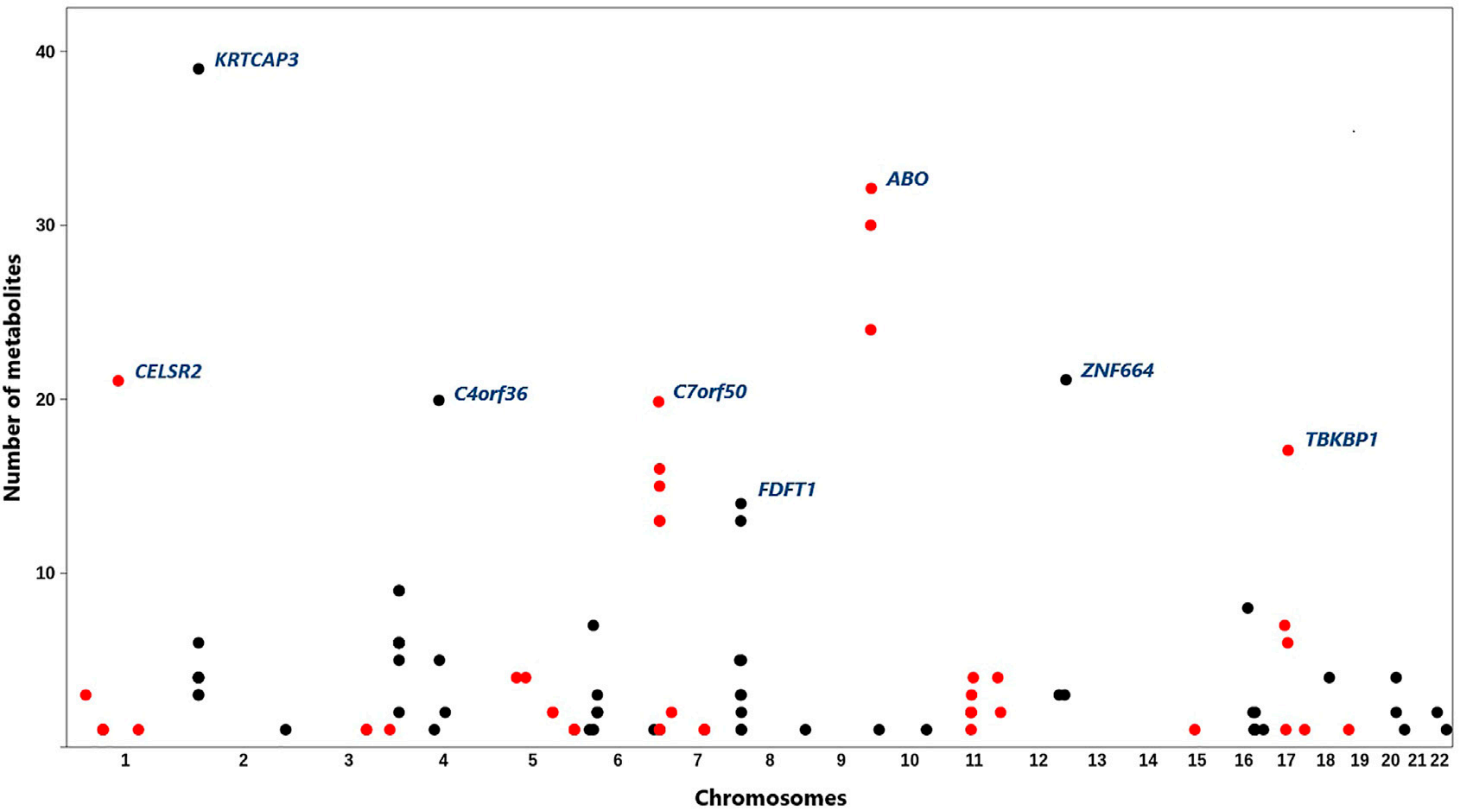
Distribution of the identified CpG sites across the genome and their associations with metabolites. *X*-axis represents physical positions of CpG sites (based on the hg19), *y*-axis indicates the number of metabolites associated with each site. A total of 107 CpG sites associated with 84 metabolites were identified. Most methylation sites were associated with more than one metabolite. CpG sites with the highest number of associated metabolites are annotated by their underlying genes (according to the ANNOVAR software). CpG sites on adjacent chromosomes are colored differently to aid viewing.

Following the analysis plan described in Figure 1, 553 pairs of epigenomic-metabolite biomarkers were detected that reached GWAS significance level (*p*-value < 5e^−8^) at both the discovery and replication stages (Supplementary Table S1). This represents 107 CpG sites associated with 84 metabolites (Figure 2) comprising 9 amino acids, 6 proteins, 66 lipids and 3 carbohydrates. A large percentage of the observed associations (N = 469, 85%) was with lipids which is higher (*p* = 0.0003, Binomial test) than expected (N = 435, 79%). This suggests epigenomics possibly plays a more important role in regulating lipids as compared to other categories of metabolites. Given that dyslipidemia contributes to a number of disorders, epigenomic intervention could theoretically be a reasonable approach toward monitoring and treating dyslipidemia.

Most methylation sites were associated with multiple metabolites; however, several CpG sites were specifically associated with one metabolite (Supplementary Table S2). For example, cg20133200 site within the phosphofructokinase gene (*PFKP*) was associated with glucose only (*β* = 0.07, P = 2e^−19^) and cg10760299 within glycine amidinotransferase (*GATM*) impacted the level of creatinine (*β* = 0.05, *p* = 1.5e^−30^). Targeting such methylation sites for therapeutic applications is notable because they show specificity. A number of CpG sites were associated with a large number of metabolites (Figure 2). For example, methylation sites within the 2p23.3 region (between *NRBP1* and *KRTCAP3*) were associated with 39 metabolites of various types. Methylation of *ABO* locus impacted 32 metabolites; whereas, a methylation site near *PSRC1* was associated with 21 lipids.

**FIGURE 3:**
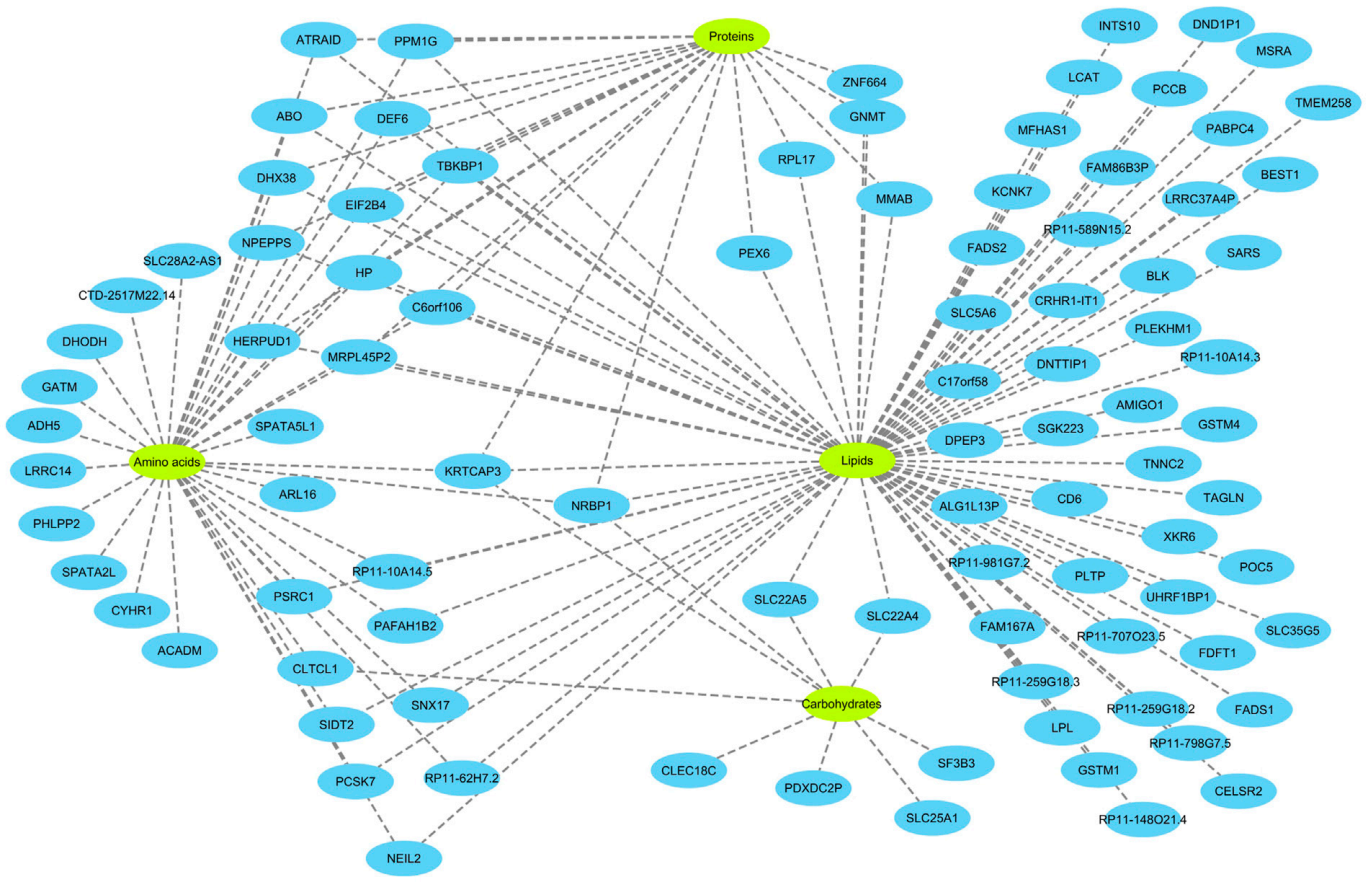
Overview of the genes identified in this study and their relations with metabolites. The analyses revealed 82 genes associated with 84 metabolites. To better visualize the findings, the metabolites were classified into four categories (amino acids, proteins, lipids and carbohydrates). 40 of the genes were specifically associated with lipids. Several genes were associated with multiple categories of metabolites. Notably, *KRTCAP3* and *NRBP1* on chromosome 2p23.3 region, that were associated with all 4 categories of metabolites. Detailed information on the nature of association of the identified genes with metabolites and underlying CpG sites are provided in the Supplementary Table S3.

Next, eQTL data from the eQTLGen consortium were integrated in order to find genes that mediate the impact of CpG sites on metabolites. Theoretically, a gene that mediates the impact of a methylation site on a metabolite must fit in the equation: CpG site → gene expression → metabolite. Namely, it must be under the regulatory impact of the methylation site and impact the level of the metabolite. Based on these criteria, 82 genes were identified (Figure 3). The majority of CpG sites were associated with one gene; however, a few were associated with a high number of genes (Supplementary Figure S1, Supplementary Table S3). By subjecting the identified genes to functional enrichment analysis, I noted they are related and share a significantly higher number of functional interactions (P<1e^−16^) than would be expected for a random set of genes of similar size, drawn from the genome (Supplementary Figure S2).

The function of a number of these genes in metabolism is well established. For example, *FADS1, FADS2, LCAT, ABO, PLTP,* and *FDFT1* are known to be involved in lipid metabolism. FADS1 and FADS2 are fatty acid desaturases. LCAT converts free cholesterol into cholesteryl ester in lipoprotein particles. PLTP functions by transferring phospholipids from triglyceride-rich lipoproteins to high-density lipoproteins. FDFT1 is an enzyme involved in cholesterol biosynthesis and MMAB helps break down certain proteins, fats, and cholesterol. Some of these genes such as *ACADM, GATM, GNMT, LCAT, MMAB, PCCB, PEX6, SLC22A4, SLC22A5,* and *SLC25A1* are known to cause inborn errors of metabolism.

Understanding the link between the identified biomarkers and the phenome is important for clinical purposes. In this regard, cardiometabolic traits are notable because they are a direct consequence of abnormalities at the metabolic level. For this purpose, I obtained GWAS summary statistics for coronary artery disease (CAD) (van der Harst and Verweij, 2018), body mass index (BMI) (Pulit et al., 2018), type 2 diabetes (T2D) (Mahajan et al., 2018), and hypertension (Source: United Kingdom Biobank) and examined their associations with biomarkers identified in this study. Below, I review the findings:

### T2D

#### PABPC4

**FIGURE 4:**
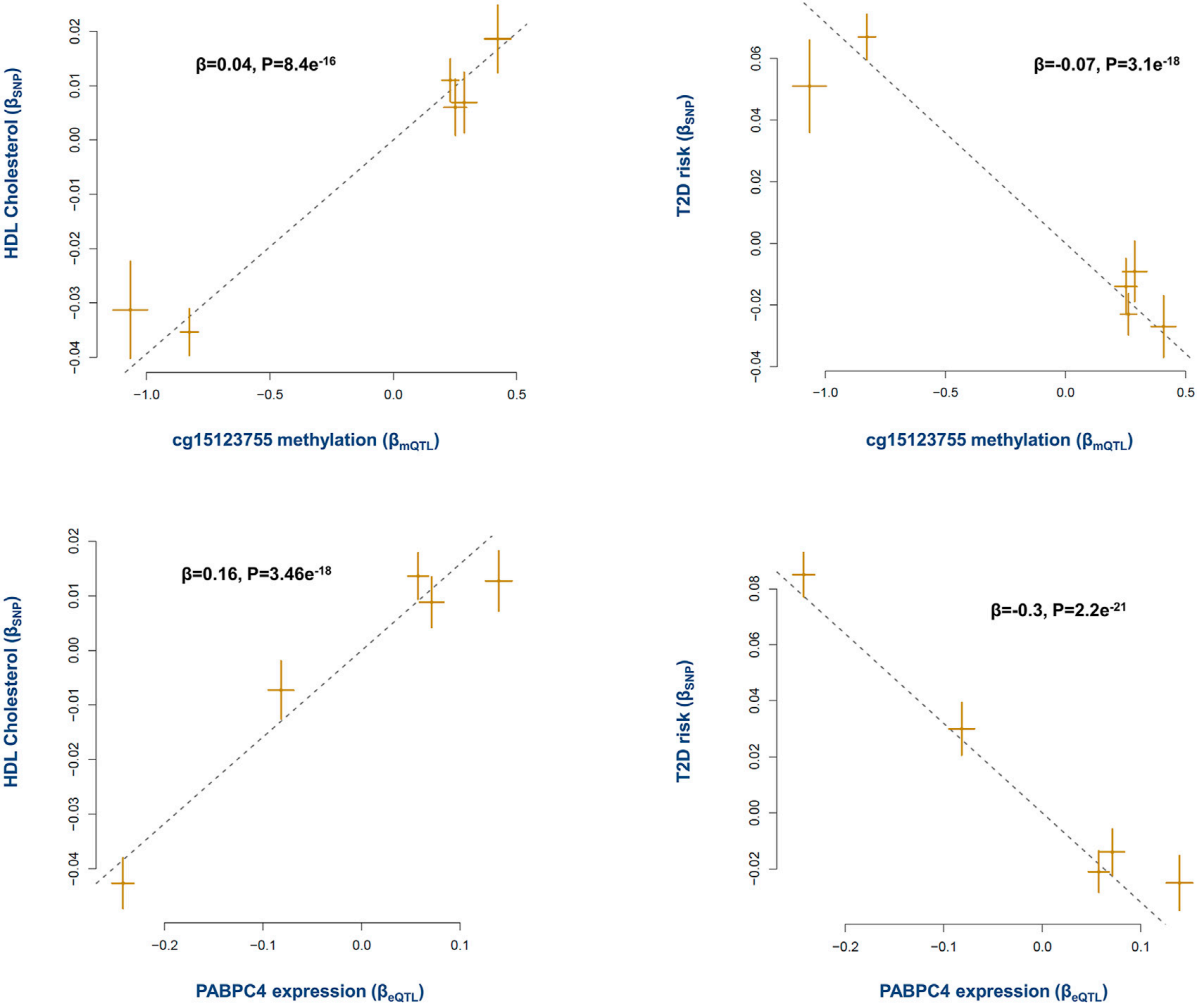
The mechanism whereby cg15123755 site impacts the risk of T2D. Higher methylation at cg15123755 site lowers the risk of T2D by changing the levels of *PABPC4* and HDL. As cg15123755 site becomes methylated the expression of *PABPC4* increases, this leads to higher level of HDL and consequently lowers the risk of T2D. Plots provide a graphical display of Mendelian randomization results. Each point on a plot represents a SNP. The x-value of the SNP is its effect size on the exposure, and the horizontal error bar represents the standard error around the effect size. The *y*-value of the SNP is its effect size on the outcome, and the vertical error bar represents the standard error around the effect size. The dashed line represents the line of best fit (i.e., a line with an intercept of 0 and slope of the effect size (*β*) from the Mendelian randomization test). A positive slope (+*β*) indicates as the level of exposure increases the level of outcome increases as well, whereas a negative slope (-*β*) indicates a negative association.

Epidemiological evidence indicates higher levels of HDL lower the risk of T2D (Femlak et al., 2017). Here the analysis revealed methylation site cg15123755 (on chromosome 1p34.3) contributes to this effect through *PABPC4* gene. The results indicated as cg15123755 site becomes methylated, the expression of *PABPC4* increases (*β* = 0.20, P = 2e^−48^, Supplementary Table S3). This in return has a positive impact on the level of HDL and consequently lowers the risk of T2D (Figure 4).

A previous report by the international knockout mouse consortium also documented *PABPC4* mutant mice show impaired glucose tolerance (GERDIN, 2010). PABPC4 is a poly(A)-binding protein as such it can impact various mRNAs. Therefore, by putting these data together, one may conclude PABPC4 targets mRNAs involved in metabolic processes. In this regard, a previous study reported depletion of PABPC4 in erythroblasts impacts a subset of mRNAs involved in developmental pathways including mRNAs critical to cell growth, and metabolism (Kini et al., 2014). Xie et al. recently reported PABPC4 regulates the level of MYC which is involved in several processes including metabolism (Xie et al., 2021).

#### 2p23.3 locus

**FIGURE 5:**
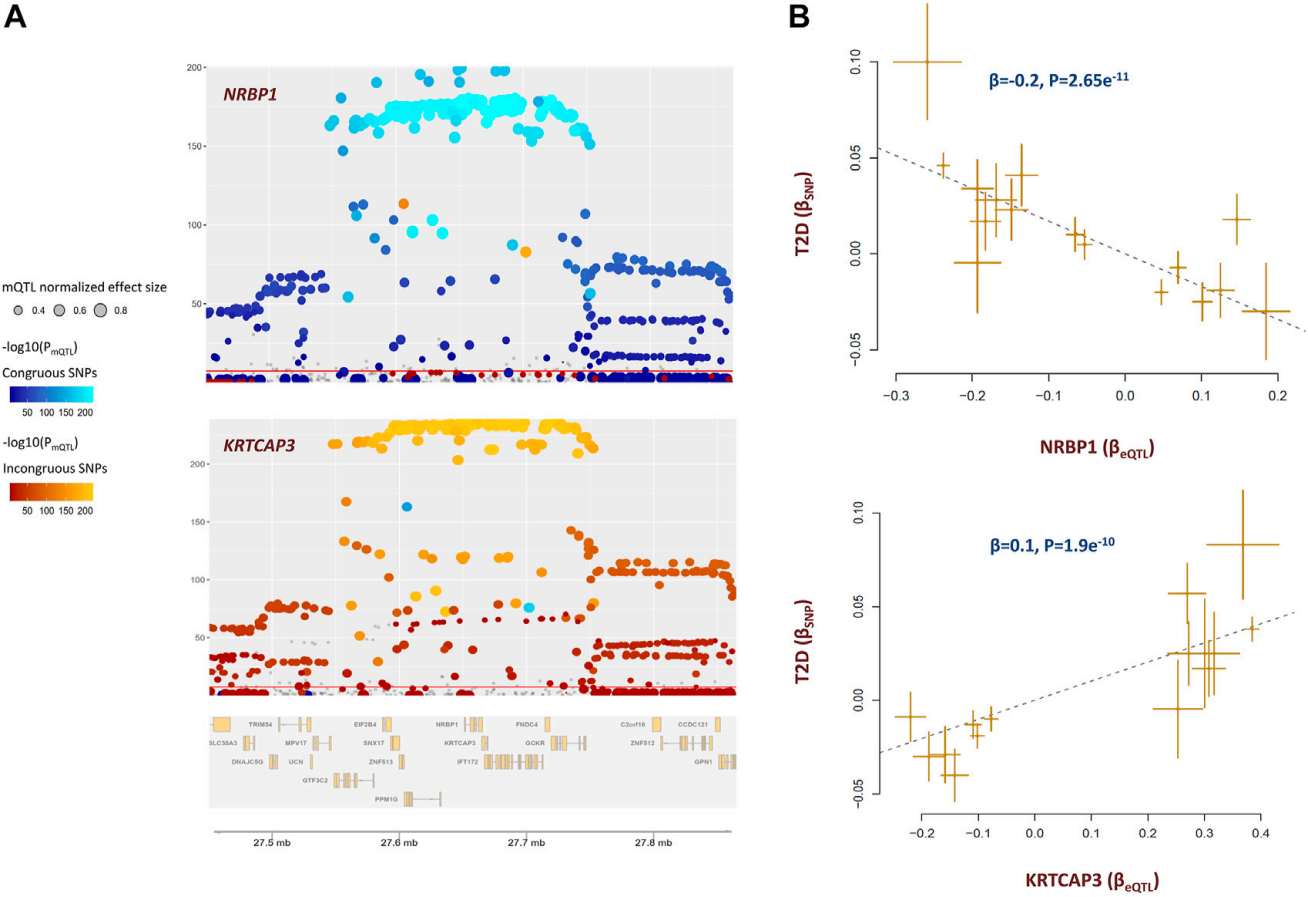
cg20102877 methylation site impacts the risk of T2D by changing the expression of *NRBP1* and *KRTCAP3*. **(A)** These plots illustrate the co-localization of eQTLs for *NRBP1, KRTCAP3 with* mQTLs for cg20102877. mQTLs for cg20102877 showed congruence with eQTLs for *NRBP1* but incongruence with eQTLs for *KRTCAP3*. This indicates higher methylation at cg20102877 increases the expression of *NRBP1* but lowers the level of *KRTCAP3* (please see Supplementary Table S3 for statistical evidence). Each point represents a SNP. The color of the point indicates its association with cg20102877 in reverse logarithmic scale. i.e., −log10(P_mQTL_). The *x*-coordinate indicates the physical position of SNP in 2p23.3 region; whereas, the y-coordinate indicates the association of SNP with gene expression in reverse logarithmic scale. The plots were generated with the eQTpLot R package. **(B)** Mendelian randomization plots indicate change in expression of these genes consequently impacts the risk of T2D. Higher expression of *NRBP1* decreases (-*β*) the risk of T2D; whereas, higher expression of *KRTCAP3* increases (+*β*) the risk of T2D. Additional information on a Mendelian randomization plot is provided in the Figure 4 description.

The methylation site cg20102877 within *KRTCAP3* was associated with T2D (Supplementary Table S4). Higher methylation at this site lowers the risk of T2D through a diverse set of metabolites. The underlying genes were *NRBP1* and *KRTCAP3* which are situated alongside each other. Both genes are under the regulatory impact of cg20102877; however, in an antagonistic pleiotropic manner. As presented in Figure 5A, mQTLs for cg20102877 showed congruence with eQTLs for *NRBP1* but not with eQTLs for *KRTCAP3*. As a result, higher methylation at cg20102877 site contributes to the risk of T2D by increasing the expression of *NRBP1* but lowering the level of *KRTCAP3* (Figure 5B).

Previous studies indicated *KRTCAP3* is implicated in food intake, adiposity, and insulin sensitivity (Szalanczy et al., 2022). Less is known about *NRBP1*, it is involved in endoplasmic reticulum to Golgi vesicle-mediated transport. It modulates the expression of uric acid transporter, ABCG2 which is involved in transport of various molecules across extra- and intra-cellular membranes (Zhang et al., 2021). Nonetheless, our findings indicate KRTCAP3 and NRBP1 are functionally related, and are possibly components of the same molecular pathway.

### CAD

#### PSRC1

**FIGURE 6:**
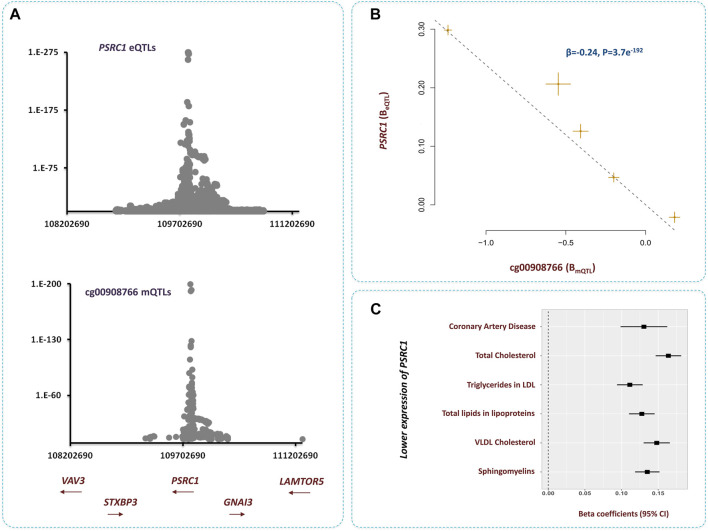
Higher methylation at cg00908766 site increases the risk of CAD through *PSRC1*-lipids path **(A)** Regional association plots for mQTLs of cg00908766, and eQTLs of *PSRC1* overlap. **(B)** MR analysis revealed, as cg00908766 becomes hypermethylated, the expression of *PSRC1* decreases. Additional information on a Mendelian randomization plot is provided in the Figure 4 description. **(C)** Lower expression of *PSRC1* contributes to higher levels of lipids and consequently higher risk of CAD.

The methylation site cg00908766 within 1p13.3 contributed to CAD by changing the expression of *PSRC1* gene (Figure 6). We noted higher methylation at this site lowers the level of *PSRC1* (*β* = −0.24, *p* = 3.7e^−192^, Supplementary Table S3) and as the expression of this gene decreases the risk of dyslipidemia and CAD increases (Figure 6). It is reported PSRC1 functions in the molecular path that mediates the impact of a TMAO-rich diet on atherosclerosis (Luo et al., 2022a). Recently, Luo et al. documented higher level of TMAO in blood lowers the expression of *PSRC1* by hypertmethylating the promoter of this gene. This consequently impairs reverse cholesterol transport and enhances cholesterol uptake and inflammation (Luo et al., 2022b). This site is also implicated in regulating the levels of several blood proteins pertinent to CAD pathogenesis (Nikpay et al., 2022). Therefore, resetting the methylation level at this site is anticipated to have a vast impact on atherosclerosis.

### Obesity

**FIGURE 7:**
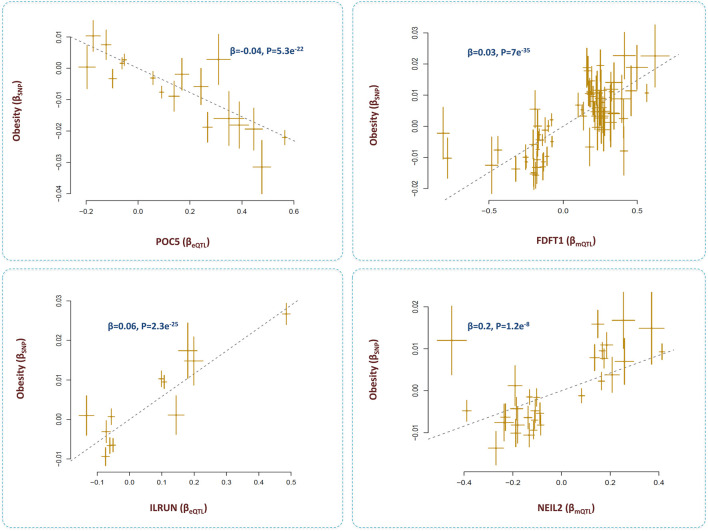
Genes that mediate the impact of methylation sites on obesity through lipid pathways. I identified methylation sites that contributed to obesity by changing the levels of several metabolites. By integrating eQTL data, the underlying genes were identified as *POC5, ILRUN, FDFT1* and *NEIL2*. Higher expression of *FDFT1, NEIL2,* and *ILRUN* was associated with higher risk of obesity; however, higher expression of *POC5* protected against obesity. Additional information on a Mendelian randomization plot is provided in the Figure 4 description.

The outcome of analyses revealed four loci that contributed to obesity through the epigenome-lipid path (Figure 7). Below, I review these loci and their relations with obesity.

#### POC5

SNPs within this gene are reported to be associated with obesity (Pulit et al., 2018). Here, I found these SNPs impact the methylation level at cg00601450 site, expression of *POC5*, and the levels of several lipid metabolites. I found as cg00601450 site becomes methylated, the expression of *POC5* increases (Β = 0.15, P = 3e^−81^) and this leads to higher levels of lipids and obesity (*β* = 0.01, P = 8e^−12^). The protein encoded by *POC5* is a component of cilium/centriole; therefore, it remains to be investigated how this protein contributes to obesity through lipids.

#### ILRUN

The outcome of analyses revealed methylation site, cg17674042 at 6p21.31 region contributes to obesity (*β* = −0.02, *p* = 4.3e^−16^) through lipids (Supplementary Table S1). The underlying gene appears to be *ILRUN* which was significantly associated with cg17674042 (*β* = −0.4, *p* = 1.2e^−50^), BMI (*β* = 0.06, *p* = 2.3e^−25^) and lipid metabolites (Supplementary Table S3).

*ILRUN*, formerly known as *C6orf106*, is a gene with cardiometabolic functions. It encodes a protein that contains a ubiquitin-associated–like domain and is shown to bind to ubiquitinylated proteins including PPARα. Mice deficient for *ILRUN* display significantly lower plasma cholesterol levels resulting from reduced liver lipoprotein production (Bi et al., 2020). The gene is also known to act as an inhibitor of antiviral and proinflammatory cytokine transcription and as a regulator of the renin-angiotensin-aldosterone system (RASS). ILRUN is considered as an inhibitor of p300/CBP transcriptional coactivators. It has been suggested that the impact of ILRUN on lipids and obesity may be *via* its transcription regulatory effect on components of RAAS (Tribolet et al., 2021).

#### FDFT1-NEIL2

We found the methylation site, cg12568669 within *FDFT1* gene contributes to obesity by changing the expression of *FDFT1* and the nearby gene *NEIL2* (Supplementary Table S4). These genes appear to be coexpressed as their eQTLs show congruence (*β* = 0.1, *p* <0.001). Lower methylation at cg12568669 contributed to higher expression of *FDFT1* (*β* = 0.4, P = 8e-118) and *NEIL2* (*β* = 0.3, P = 8e-110) and this, in turn, contributed to higher BMI by impacting the levels of creatinine and unsaturated fatty acids (Figure 7, Supplementary Table S4). A previous study in a sample of US Caucasians found SNP, rs7001819 located in the intergenic region between *NEIL2* and *FDFT1* shows association with BMI (Liu et al., 2008). We noted this SNP is also an eQTL for both NEIL2 and FDFT1 (based on eQTLGen data).

*FDFT1* is a gene with well-established metabolic function, it encodes an enzyme of the mevalonate pathway that is responsible for producing various metabolites including cholesterol. Mevalonate pathway is vital to maintain adipocyte survival and obesity is essentially the outcome of an increase in adipose tissue mass (Yeh et al., 2018). *NEIL2* encodes an enzyme involved in base excision repair of DNA damage by oxidation. Unrepaired DNA damages or defects in DNA repair pathways can lead to cellular dysfunction, and metabolic disturbances. Mutations of genes related to DNA repair are reported to cause obesity (Komakula et al., 2018; Włodarczyk and Nowicka, 2019).

### Hypertension

The analyses revealed positive associations between the expression of *KCNK7*, *FDFT1-NEIL2* locus and hypertension (Supplementary Table S3).

#### KCNK7

The methylation site cg21033440 upstream of *KCNK7* contributed to hypertension (*β* = 0.01, *p* = 3.8e^−11^, Supplementary Table S4) by increasing the expression of this gene (*β* = 0.14, *p* = 8.9e^−8^, Supplementary Table S3). *KCNK7* is a member of potassium channels known as weak inward rectifiers. They conduct outward K^+^ currents that maintain the resting membrane potential and modulate action potential repolarization. A member of this gene family, *KCNK6* is known to contribute to hypertension (Pandit et al., 2014). It remains to be investigated how *KCNK7* contributes to hypertension through lipids. However, inward-rectifier potassium channels are known to be regulated by a variety of different stimuli including lipid metabolites.

#### NEIL2 and FDFT1

The methylation site cg12568669 which was reported in the previous section to be associated with obesity also impacted blood pressure in a similar pattern, namely, by changing the expression of *NEIL2* and *FDFT1* genes. A hypomethylated cg12568669 site, was associated with higher expression of *NEIL2* (B = −0.3, *p* = 8.3e-110) and *FDFT1* (B = −0.4, *p* = 8.3e-118) and these changes in expression consequently raised blood pressure by impacting the levels of fatty acids and triglycerides (Supplementary Table S4). The association between *FDFT1* and hypertension could be attributed to its involvement in cholesterol biosynthesis. Cholesterol plaque causes arteries to narrow and as a result, blood pressure increases. Furthermore, hypercholesterolemia is associated with higher oxidative DNA damage and increased level of DNA repair (Martinet et al., 2001). This could also explain why *FDFT1* and *NEIL2* have coordinated expression.

### Connection with lifestyle factors

CpG sites undergo change in response to an individual’s lifestyle and consequently impact downstream processes such as metabolome. Therefore, it is important to elucidate the relation of CpG sites with lifestyle factors. Here, I searched the content of publicly available EWAS databases (Li et al., 2018; Battram et al., 2022) and identified a number of lifestyle habits (Supplementary Table S5) that influence the CpG sites presented in this study. Such findings could aid personalized medicine approaches. For example, according to data in Supplementary Table S1, *ABO* appears to play a key role in metabolism. Therefore, if a person has a cardiometabolic disorder and shows high methylation at *ABO* locus. The main trigger could be exposure to smoking or pesticides (Supplementary Table S5). Conversely, cg10760299 within *GATM* gene was only associated with creatinine. Therefore, if a person has abnormal creatinine level and abnormal methylation level at cg10760299, the drinking behavior could be the cause. By recording and indexing this information in a database, in the long term, it is possible to give personalized lifestyle recommendations to an individual based on the degree of alterations in her/his epigenome, in order to keep the body on track and prevent health problems.

## Discussion

Over the past two decades, numerous studies have been conducted to find genomic variants (SNPs) that contribute to phenotypes (traits and functional features). Findings from these studies have expanded our knowledge in several domains including disease diagnosis, risk assessment, and molecular biology of diseases (Nikpay et al., 2017; Uffelmann et al., 2021). The outcome of these studies has provided the research community with an unprecedented volume of data known as GWAS summary statistics that outline the nature of association between SNPs and phenotypes. These data are now being combined to understand the nature of association between functional features and the traits (Nikpay et al., 2020; Akiyama, 2021; Nikpay and McPherson, 2021). This study is another attempt in this direction. By integrating epigenome and metabolome data, here I reported 107 CpG sites associated (P<5e^−8^) with 84 metabolites through a two stages, discovery/replication analysis; furthermore, by including eQTL data, I investigated genes that mediate the impact of CpG sites on metabolites.

The human metabolome database (HMDB) is a publicly available database containing detailed information about small molecule metabolites found in the human body (Wishart et al., 2021). Several of the genes identified in the current study were also indexed in HMDB (version 5.0) database. These include *ACADM, GSTM1, GSTM4, SARS, EIF2B4, PPM1G, SLC5A6, PCCB, ADH5, SLC22A4, SLC22A5, DEF6, GNMT, BLK, FDFT1, MSRA, ABO, CD6, BEST1, FADS1, FADS2, MMAB, GATM, LCAT, DHX38, HP, PHLPP2, PLEKHM1, PLTP, CLTCL1,* and *SLC25A1*. A number of CpG sites identified in this study are previously reported to impact the blood proteome. This includes cg00908766 (*PSCR1*), cg21160290 (*ABO*), cg26840970 (*HP*), cg07404485 (*PON1*), cg21280719 (*GNMT*) (Nikpay et al., 2022). Secolin et al. (Secolin et al., 2021) reported a region on chromosome 8 (8p23.1) that has been positively selected in the Brazilian population because it had offered metabolic advantage in the early stages of population admixture. I identified a number of methylation sites within this region that impacted the levels of several metabolites, notably lipids (Supplementary Table S1).

The identified biomarkers were tested with regard to cardiometabolic traits by including GWAS summary statistics of these phenotypes into the analyses (Figure 1). Nine genes were identified that contributed to cardiometabolic disorders by mediating the impact of CpG sites on metabolites. The functions of a number of these genes in metabolism have been documented including *ILRUN, FDFT1,* and *PSRC1*; however, the metabolic functions of *PABPC4, NRBP1, KRTCAP3, POC5, NEIL2* and *KCNK7* remain less understood and further research is required. Previously, Pividori et al. (Pividori et al., 2020) used a Bayesian colocalization approach to construct a database (PhenomeXcan) of causal gene-trait associations by integrating transcriptomic data from 49 tissues in GTEX and GWAS summary statistics of phenotypes. By examining their findings, I found concordance with their results. As presented in Supplementary Table S6, the genes identified in the current study showed consistent and significant direction of association with cardiometabolic traits in the PhenomeXcan database as well. I noted genes under the regulatory impact of a CpG site are not consistently contributing to a trait. For example, cg00908766 on 1p13.3 chromosome band impacted the levels of 5 genes but among them only *PSRC1* contributed to the risk of CAD. Therefore, considering the pleiotropic effect of a CpG site is important in studies that aim to target an epigenomic site.

To assess the contribution of biomarkers identified in this study to other phenotypes. Data from this study was made publicly available as a Unix package that can test the association between biomarkers and a phenotype. This is important because phenome is diverse and vast and testing the association between the identified biomarkers and phenotypes is cumbersome. A detailed user guide of how to conduct a search is provided on the corresponding GitHub page (please see the data availability section).

In summary, a search can be executed as:

bash wrapper.sh ID_1_ ID_2_

Where ID_1_ is the metabolite identifier and ID_2_ is the phenotype identifier from the OpenGWAS database (Elsworth et al., 2020) which is a repertoire of GWAS summary data for various phenotypic features. A search example would be:

bash wrapper.sh met-d-Total_FA ieu-b-30

Which investigates the association between fatty acid levels and white blood cell count (WBC) after obtaining the relevant data from the OpenGWAS. The results indicate higher levels of fatty acids contribute to higher values of WBC (*β* = 0.04, *p* = 6.3e^−13^); furthermore, it reveals cg12568669 as a methylation site that contributes to this effect (Supplementary Table S7).

Cardiovascular disease (CVD) in its common form is age dependent. A likely mechanism in this regard is epigenomic modifications because the sequence of DNA remains stable throughout life; however, it actively undergoes modifications at the epigenome level. Findings from GWAS studies also indicate SNPs contributing to cardiovascular disease are enriched in epigenomic sites associated with transcriptional activity (Nikpay et al., 2017). As it was reviewd before (Napoli et al., 2012), understanding the epigenetic bases of CVD could provide novel and early markers for diagnosis. This is important because CVD progresses gradually and epigenomic biomarkers allow the detection of abnormalities years before the manifestation of major cardiometabolic outcomes. Furthermore, epigenomic modifications are reversible, therefore, if an abnormality is observed, as described in the results section (Supplementary Table S5), a change in lifestyle can provide a remedy to prevent a disease before establishing its roots (epigenomic signature) (Bacalini et al., 2014; Quach et al., 2017; Gensous et al., 2019). Therefore, more research with regard to the interplay of lifestyle, epigenomics and diseases can provide a solid foundation for personalized preventive medicine.

Several epigenome-editing therapeutics are already in use to treat complex disorders; however, the major drawback associated with them is non-specificity and therefore undesirable side effects; because they target epigenome-editing enzymes such as DNA methyltransferase that impacts the expression of various genes. The newly developed CRISPR-based epigenome editing technology circumvents this issue because it enables the epigenome-editing enzyme to bind specifically to the site of interest (Nakamura et al., 2021; Ansari et al., 2022). Findings from this study indicate CpG sites are not equal regarding their impact on a process and therefore care should be taken in choosing the correct sites for editing. While some sites showed specificity and only impacted a particular type of a metabolite (e.g., cg10760299 within *GATM* gene). There were also sites that impacted the levels of numerous metabolites (e.g., cg21160290 within *ABO* gene).

Mendelian randomization is traditionally used to identify risk factors for a disease. Here, this approach was used to obtain molecular insight by understanding the connection between biomarkers and phenotypes. MR appears to be more suited for this purpose because a biomarker is under the regulatory impact of fewer SNPs as compared to a complex trait. As such MR is less likely to suffer from weak-instrument bias when the exposure (outcome) is a biomarker. As compared to gene knockout experiments, MR provides a non-invasive path to understand the function of a locus directly in human (Nikpay et al., 2021; Nikpay and McPherson, 2021). Moving in this direction requires generating GWAS data for a more diverse set of functional features and ultimately creating a web portal that outlines biomarkers behind a disease and their connections for downstream applications.

In this study, stringent statistical criteria were applied at each step to lower the likelihood of false positives. In addition, the inclusion of data from studies with lower sample sizes, also impacts the power of the analyses. Therefore, biomarkers identified in this study must be considered as low-hanging fruits. Future studies that utilize data from larger studies will be able to provide a better picture of the epigenomics of metabolome.

In summary, this study reports the outcome of a genome-wide search for methylation sites that regulate the metabolome. The analyses revealed 107 CpG sites associated with 82 metabolites; furthermore, by including eQTL data, 82 genes were identified that mediated the impact of methylation sites on metabolites. By integrating these findings with GWAS data for T2D, CAD, obesity and hypertension, 9 genes were found that contributed to these traits through metabolic pathways. I also described a notion whereby measuring changes at the epigenome level and adjusting lifestyle accordingly could provide a path for early diagnosis and prevention of disorders. Findings from this study are publicly available as a freeware that allows investigating the contribution of the identified biomarkers to other phenotypes.

## Data Availability

GWAS summary statistics for metabolites are available from: https://gwas.mrcieu.ac.uk/datasets/?gwas_id__icontains=met-d, mQTL data was obtained from: https://yanglab.westlake.edu.cn/software/smr/#mQTLsummarydata, eQTL data was obtained from: https://www.eqtlgen.org/trans-eqtls.html, Data, instructions, and shell scripts to carry out the analyses are available from: https://github.com/mnikpay/epimetabolome-to-phenome.git.
