# Supplementary figures and images for: Genome-wide search identified DNA methylation sites that regulate the metabolome
Source: Front Genet. 2023 May 18;14:1093882. doi: 10.3389/fgene.2023.1093882 (PMC10233745; doi:10.3389/fgene.2023.1093882)

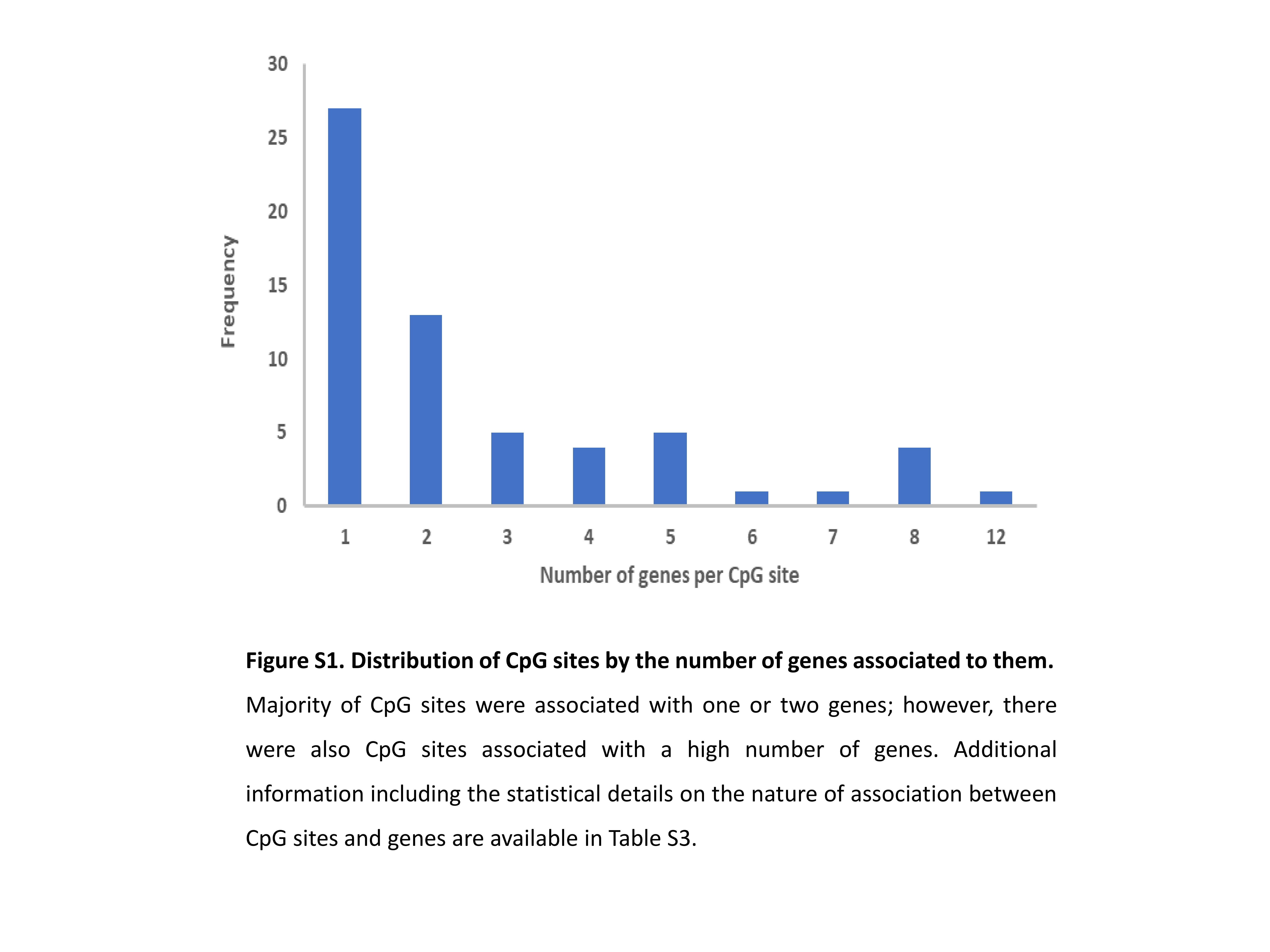

Supplement: Supplementary file 1 [file Image1.JPEG]

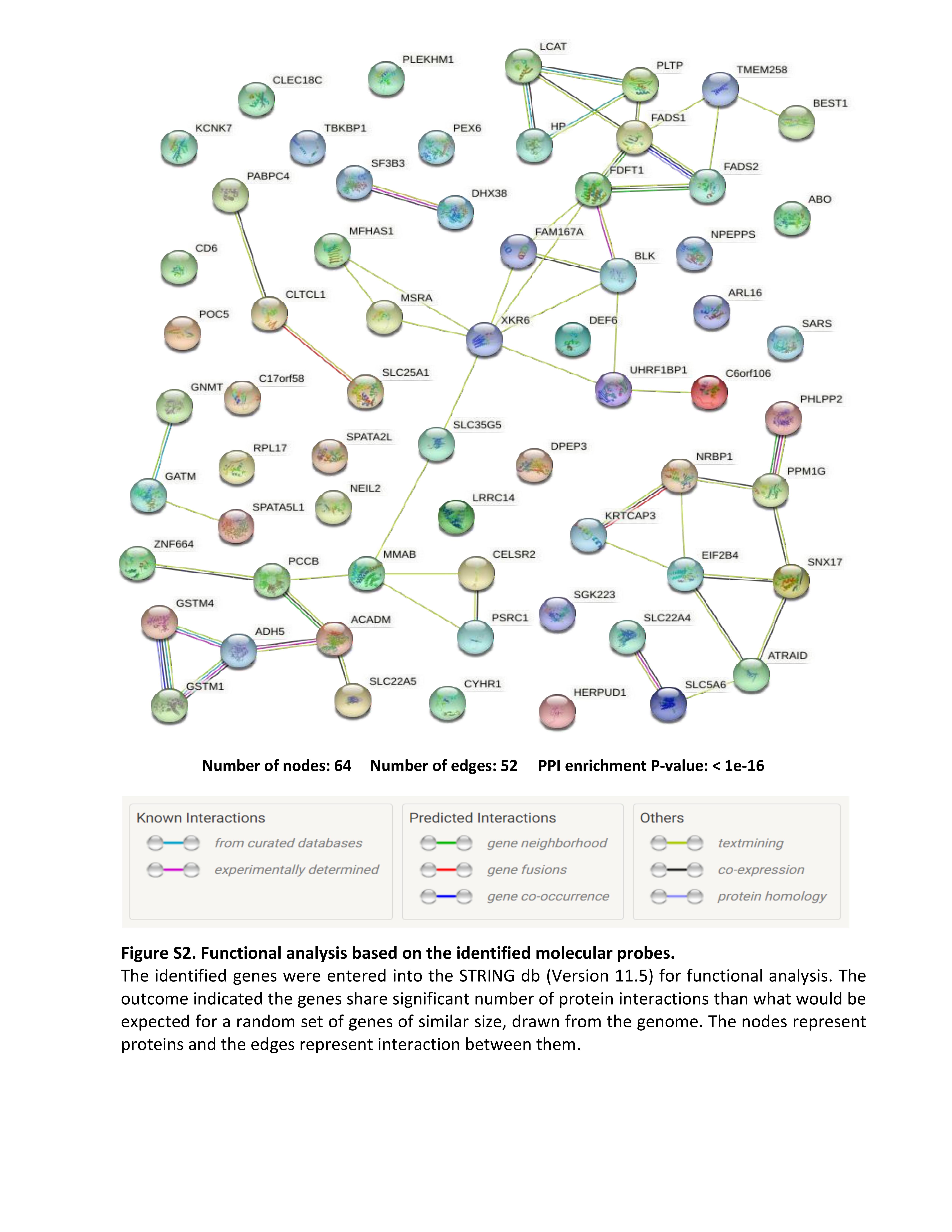

Supplement: Supplementary file 2 [file Image2.JPEG]
